# The Efficacy of Guanxinning Injection in Treating Angina Pectoris: Systematic Review and Meta-Analysis of Randomized Controlled Trials

**DOI:** 10.1155/2013/282707

**Published:** 2013-03-24

**Authors:** Yongliang Jia, Siu-wai Leung, Ming-Yuen Lee, Guozhen Cui, Xiaohui Huang, Fongha Pan

**Affiliations:** ^1^State Key Laboratory of Quality Research in Chinese Medicine, Institute of Chinese Medical Sciences, University of Macau, Macao SAR, China; ^2^School of Informatics, University of Edinburgh, Edinburgh EH8 9AB, UK

## Abstract

*Objective*. The randomized controlled trials (RCTs) on Guanxinning injection (GXN) in treating angina pectoris were published only in Chinese and have not been systematically reviewed. This study aims to provide a PRISMA-compliant and internationally accessible systematic review to evaluate the efficacy of GXN in treating angina pectoris. *Methods*. The RCTs were included according to prespecified eligibility criteria. Meta-analysis was performed to evaluate the symptomatic (SYMPTOMS) and electrocardiographic (ECG) improvements after treatment. Odds ratios (ORs) were used to measure effect sizes. Subgroup analysis, sensitivity analysis, and metaregression were conducted to evaluate the robustness of the results. *Results*. Sixty-five RCTs published between 2002 and 2012 with 6064 participants were included. Overall ORs comparing GXN with other drugs were 3.32 (95% CI: [2.72, 4.04]) in SYMPTOMS and 2.59 (95% CI: [2.14, 3.15]) in ECG. Subgroup analysis, sensitivity analysis, and metaregression found no statistically significant dependence of overall ORs upon specific study characteristics. *Conclusion*. This meta-analysis of eligible RCTs provides evidence that GXN is effective in treating angina pectoris. This evidence warrants further RCTs of higher quality, longer follow-up periods, larger sample sizes, and multicentres/multicountries for more extensive subgroup, sensitivity, and metaregression analyses.

## 1. Introduction

Ischemic heart disease (IHD) is a major cause of death and global healthcare burden [1]. Angina pectoris, a symptom of IHD, is a severe chest pain due to ischemia of the heart muscle, during obstruction or spasm of the coronary arteries [2]. In the United States, IHD accounts for 26.6% of all deaths in 2005, with an age-adjusted male-to-female mortality ratio of 1.5 [3]. The morbidity and mortality of angina in middle-aged and elderly people were ranked the top among all common diseases in China [4]. Three categories of conventional Western medicine including nitrates (e.g., isosorbide mononitrate), beta-receptor blockers (e.g., atenolol), and calcium channel blockers (e.g., amlodipine) are commonly used in treating angina [3]. 

 Guanxinning injection (GXN, also known as Danshen Chuanxiong Injection) comprises extracts from two well-known traditional Chinese medicines Danshen (Salvia miltiorrhiza) and Chuanxiong (Ligustrazine, Ligustium Wallichii Franch) [5]. Danshen and its active compounds tanshinones and isotanshinones have bioactivities against myocardial ischemia, inflammation, and angiotensin-converting enzyme [6]. Chuanxiong and its active compounds tetramethylpyrazine and ferulic acid can dilate coronary arteries, increase myocardial oxygen, and decrease platelet aggregation and thrombosis [7].

 GXN was tested to be more effective than nitrates [8], beta-receptor blockers [9], and calcium channel blockers [10] in treating angina. Since the launch of GXN (2002) and prior to this study, there has been only one systematic review, which is not compliant with PRISMA [11] and includes only nine randomized controlled trials (RCTs) published in Chinese between 2002 and 2010 [12]. The methods and results of quality assessment of the included RCTs were not clearly reported in the systematic review. Sensitivity and subgroup analyses were missing. Hence, this study aims to provide an internationally accessible, comprehensive, and timely systematic review and meta-analysis in compliance with PRISMA to assess the efficacy of GXN as a monotherapy and combined therapy with conventional Western or Chinese medicines in treating angina pectoris.

## 2. Methods

The procedures of this systematic review and meta-analysis were conducted in accordance with the PRISMA guideline [11], including the search and selection of studies, data extraction from the studies, and meta-analysis (overall, subgroup, sensitivity, publication bias, and metaregression analysis). 

### 2.1. Search Strategies

RCTs published on the efficacy of GXN in treating angina pectoris were retrieved from major bibliographical databases including Medline, PubMed, Cochrane Library, ScienceDirect, Embase, China National Knowledge Infrastructure (CNKI), WanFang Data, China Master Theses Full-text Database (CMTD), and China Doctor Dissertations Full-text Database (CDMD) between the inception dates of databases and 2012 (last search on 18 March 2012). A simple search strategy, that is, searching for the keywords “Guanxinning” or “danshen chuanxiong” or “danshenchuanxiong,” was used to search all fields. For instance, the search in WanFang Data using the keyword “Guanxinning” found 196 records and “danshen chuanxiong” found 17 records and “danshenchuanxiong” found none. Exact search strategies and query syntax for specific databases were customized according to the same strategy. 

### 2.2. Study Selection

Inclusion criteria for each study were (a) the participants were suffering from and being treated for angina pectoris; (b) the study was claimed as an RCT; (c) the study compared the efficacy of GXN with conventional (Western and Chinese medicine) drugs. Exclusion criteria were (a) the study was a duplicated or redundant publication and (b) the study did not include symptomatic improvement as a major outcome.

**Figure 1:**
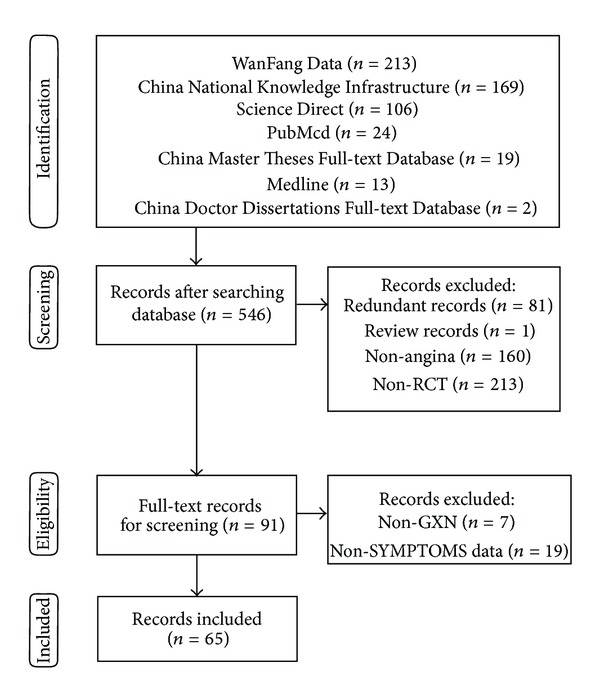
Process of searching and screening studies.

Two reviewers (Y. Jia and F. Pan) independently searched the databases and selected studies according to the inclusion and exclusion criteria. Disagreements between reviewers were resolved by consensus after discussion.  Figure 1 shows a flow diagram of study selection. 

### 2.3. Data Extraction

Two reviewers (Y. Jia and F. Pan) independently extracted data items, including (a) years of publication; (b) numbers of authors; (c) follow-up periods; (d) baseline characteristics of participants between groups; (e) sample sizes; (f) outcome measures; (g) dosages and follow-up periods; (h) type of angina; (i) frequencies of adverse events (AE); and (j) the type of angina.

### 2.4. Quality Assessment of Included Studies

Two reviewers (Y. Jia and F. Pan) independently assessed the quality of the included studies according to the Jadad scale [13], its refined version the *M* scale [14], and the Cochrane Collaboration's tool for assessing risk of bias [15]. The Jadad scale focused on three criteria including “randomization,” “blinding,” and “dropouts” for assessing the quality of RCT. The *M* scale added two criteria “baseline comparison of participants” and “adverse event report” on top of the Jadad scale. The Cochrane Collaboration's tool for assessing risk of bias includes “random sequence generation,” “allocation concealment,” “blinding of participants and personnel,” “blinding of outcome assessment (patient-reported outcomes),” “blinding of outcome assessment (SYMPTOMS),” “incomplete outcome data addressed,” “reporting bias,” and “other sources of bias.”

### 2.5. Criteria for Symptomatic and ECG Improvements

Effective symptomatic improvements should achieve at least 50% (basic) or 80% (significant) reduction in frequency of feeling angina chest pain [16]. Effective ECG improvements should achieve (a) at least 0.05 mV lowering at ST segment in ECG (basic) or (b) nearly normal (significant) ECG during an exercise test according to the International Society and Federation of Cardiology/World Health Organization [16].

### 2.6. Meta-Analysis

Effect sizes were represented by odds ratios (ORs) [17] and their 95% confidence intervals (CI) [18]. Overall meta-analysis and subgroup analysis employed the random-effects model for conservative generalizability. Heterogeneity among studies was assessed by Chi-squared (*χ* ^2^) and I-squared (*I* ^2^) tests [19]. 

### 2.7. Subgroup and Sensitivity Analyses

Subgroup analysis was conducted to evaluate the overall effects in the subgroups according to years of publication (≤2008 or >2008), numbers of authors (1 or >1), follow-up periods (≤14 days or >14 days), sample sizes (<mean sample size or ≥mean sample size), quality scores of the studies (<mean or ≥mean), different type of angina, and different daily dosage of GXN. The overall effects were also analyzed in subgroups of GXN for monotherapy and adjunctive therapy. Sensitivity analysis was carried out according to different criteria outcomes (basic or significant) in SYMPTOMS and ECG and excluding studies with maximum GXN dosage to assess their influence on the overall effect sizes. The Mann-Whitney-Wilcoxon test was used to compare two subgroups. The Kruskal-Wallis test and the Bonferroni correction were used to compare multiple subgroups. Kendall correlation between ORs of symptoms and ECG was performed. 

### 2.8. Metaregression and Risk of Bias across Studies

Funnel plots [20], Begg's test [21], and Egger's test [22] were employed to assess publication bias. Trim-and-fill method [23] was conducted to identify and correct the funnel plot asymmetry arising from publication bias. Metaregression [24] was conducted to find the possible relationship between the overall effects and the factors such as sample sizes, follow-up periods, *M* scores, and years of publication. 

### 2.9. Adverse Events

Information about adverse events (AEs) of RCTs, including nonreported adverse events and types and frequency of adverse events reported, was tabulated and analyzed by basic statistics. 

### 2.10. Statistical Analysis

All data analyses, including meta-analysis, forest plot generation, funnel plot generation, metaregression, Kendall correlation, Mann-Whitney-Wilcoxon test, Kruskal-Wallis test, Begg's test, and Egger's test, were performed using statistical software R [25] and its “metafor” package for meta-analysis. *P* values lower than 0.05 were considered statistically significant.

## 3. Results

### 3.1. Study Selection

Figure 1 depicts the process of study selection. The search of bibliographical databases found 401 records, including 196 records from WanFang Data, 162 records from CNKI, 19 records from CMTD, 11 records from ScienceDirect, 6 records from Medline, 5 records from PubMed, and 2 records from CDMD. According to prespecified selection criteria as described in Methods, 65 studies [26–90] were included for further quality assessment and meta-analysis. 

### 3.2. Study Characteristics

Table 1 lists the main characteristics of the included studies. All included studies were published in the Chinese language between 2004 and 2011 with a total of 6064 participants. The mean sample size was 93.3 (median: 88.0; 95% CI: [56.5, 130.1]). The follow-up periods were between 1 and 30 days. GXN was compared with the conventional treatments in the included RCTs. Drugs in control group mainly included nitrates, beta-receptor blockers, calcium channel blockers, angiotensin-converting enzyme inhibitors, and some conventional Chinese medicinal products for treating heart disease. Fifty-nine out of 65 RCTs employed GXN plus the conventional treatments in the treatment group while the conventional treatments were employed in control group. Dosage details were listed in Supplementary Table  1 in the Supplementary Material available online at http://dx.doi.org/10.1155/2013/282707. For outcome measures, all 65 included studies reported symptomatic (SYMPTOMS) changes while 38 studies also reported ECG changes.

### 3.3. Quality Assessment of Included Studies

Table 2 shows the results of quality assessment according to the Jadad scales, *M* scales, and the Cochrane Collaboration's tool. According to the Jadad scale (with a possible range between 0 and 5 points), 63 studies of all included studies scored 2 with two items “randomization” and “dropouts,” one study [34] scored 3, and one study [47] scored 4. According to the *M* scale, six studies scored 2, three studies scored 2.5, 30 studies scored 3, 24 studies scored 4, and 2 studies scored 5. Fifty included studies reported baseline comparison of participants in experiment and control groups. Thirty-one studies did not report adverse events. Three studies reported types of adverse events. Thirty-one studies reported types and numbers of adverse events. The assessment results of the Cochrane Collaboration's tool showed (1) low risk of bias in random sequence generation for selection bias, blinding of outcome assessment (SYMPTOMS) for detection bias, and incomplete outcome data addressed for attrition bias, (2) high risk of bias in allocation concealment for selection bias, blinding of participants and personnel for performance bias, blinding of outcome assessment (patient-reported outcomes) for detection bias, and reporting bias for selecting reporting, and (3) unclear risk of bias in other sources of bias for other bias. 

### 3.4. Overall Effects of Included Studies

**Figure 2:**
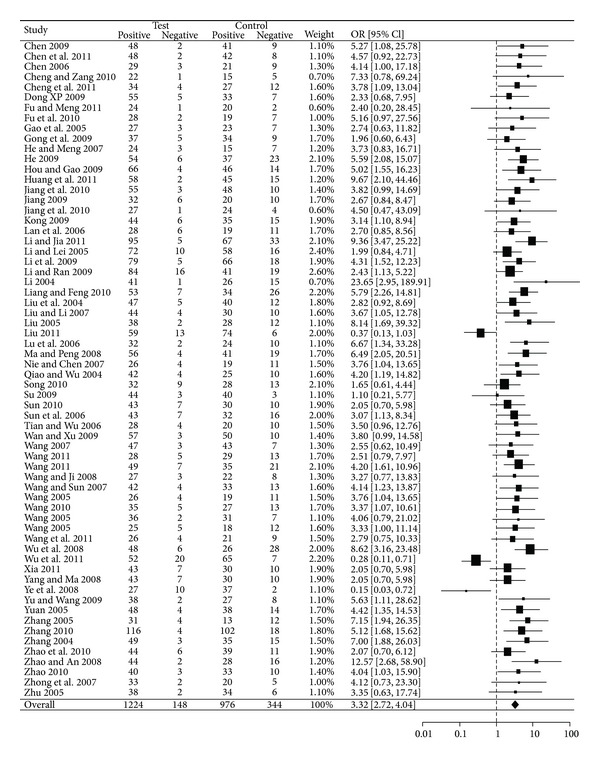
Forest plot of outcome measure SYMPTOMS.

**Figure 3:**
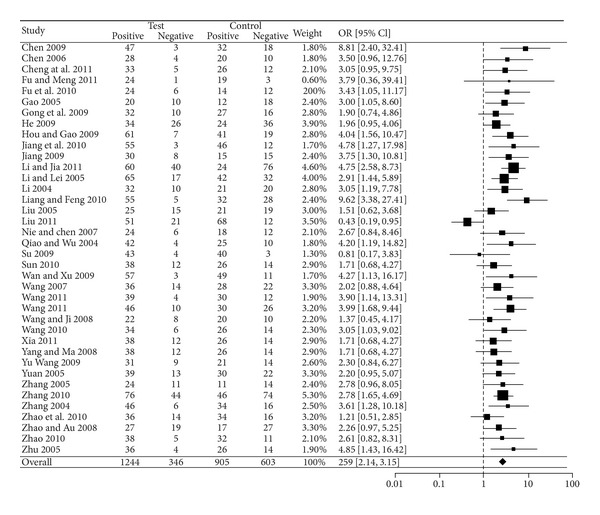
Forest plot of outcome measure ECG.

As shown in Figure 2 and Table 3, the overall OR of SYMPTOMS was 3.32 (95% CI: [2.72, 4.04], *Z* = 11.93, *P* < 0.0001) with significant heterogeneity (tau = 0.23, *I* ^2^ = 37%, *P* = 0.0030) among the 65 studies with SYMPTOMS outcome.  Figure 3 and Table 4 show that the overall OR of ECG was 2.59 (95% CI: [2.14, 3.15], *Z* = 9.68, *P* < 0.0001) with nonsignificant heterogeneity (tau = 0.11, *I* ^2^ = 32%, *P* = 0.0539) among the 38 studies with ECG outcome. Both ORs (SYMPTOMS and ECG) indicated that GXN was more effective than the drugs in control group in treating angina pectoris. The Kendall correlation between SYMPTOMS and ECG in ORs was statistically significant (tau = 0.2644; *P* = 0.0200). 

### 3.5. Subgroup Analysis

ORs of the subgroups in both SYMPTOMS (Table 3) and ECG (Table 4) were compared based on the study characteristics including *M* scores (≤3 or >3), sample sizes (<93 or ≥93), number of authors (1 or >1), years of publication (before or after January 1, 2008), reports of trial dates (yes or no), baseline comparison of participants (yes or no), reports of adverse events (yes or no), follow-up periods (≤14 days or >14 days), GXN daily dosages (<20 mL, 20 mL, >20 mL), different angina types, and different treatments including GXN monotherapy versus control treatment, GXN + control versus control, and GXN mixed treatment + control versus control. There was no statistically significant difference between ORs of these subgroups. 

### 3.6. Sensitivity Analysis

When the improvement criteria were raised to the significant level from the basic level, the overall results remained effective (i.e., OR > 1) and statistically significant. The OR of overall SYMPTOMS decreased from 3.32 to 1.75 (95% CI: [1.54, 1.98], *Z* = 8.65, *P* < 0.0001). The OR of overall ECG decreased from 2.59 to 1.84 (95% CI: [1.59, 2.14], *Z* = 8.06, *P* < 0.0001). There was a statistically significant correlation between the changes in ORs of SYMPTOMS and ECG outcomes (tau = 0.2971, *P* = 0.0089). When study [33] with maximum GXN dosage was excluded, there was no statistically significant difference between ORs of groups in both SYMPTOMS and ECG data.

### 3.7. Metaregression

Table 5 shows the results of metaregression between log OR and study characteristics. There seemed to be no statistically significant relationship between GXN's efficacy and study characteristics, except that follow-up periods made a significant difference (*P* = 0.0093) on the log OR with ECG data. 

### 3.8. Risk of Bias Across Studies

**Figure 4:**
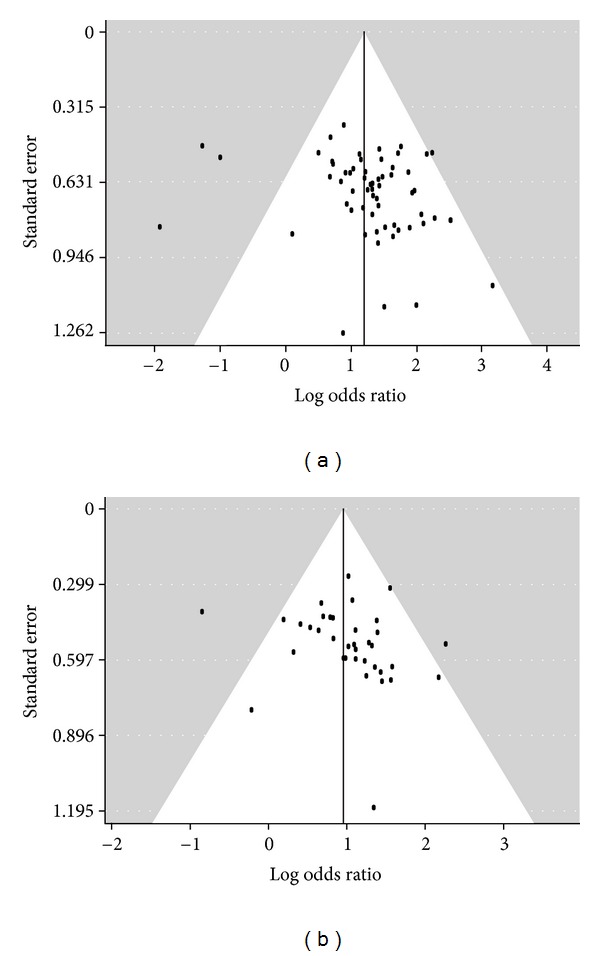
Funnel plots of (a) the included studies with SYMPTOMS data and (b) the included studies with ECG data.

Visual assessment of funnel plots (Figure 4) found obvious asymmetry, indicating that there were publication biases in the results of both SYMPTOMS and ECG. Egger's test (SYMPTOMS: *t* = 2.0555, *P* = 0.0440; ECG: *t* = 0.9358, *P* = 0.3556) and Begg's test (SYMPTOMS: *z* = 0.1898, *P* = 0.0257; ECG: *z* = 0.2571, *P* = 0.0236) detected statistically significant publication biases. Trim-and-fill method found that there were 24 missing studies for SYMPTOMS and 13 missing studies for ECG on the left side of the corresponding funnel plots.

### 3.9. Adverse Events

As shown in Table 6, the most frequently reported adverse event of GXN was headache. All adverse effects were minor or well tolerated as they did not cause dropouts except in one study [31] where six participants dropped out because of the adverse effects. Headache, epigastria discomfort, and palpitation were noted as the top three adverse effects of drugs in control group. Adverse effects of GXN were less than those of control drugs in the number of types, severity, and frequency.

## 4. Discussion

This study provides the first comprehensive, up-to-date, and PRISMA-compliant systematic review on the efficacy of GXN in treating angina pectoris. Among 65 included RCTs with 6064 participants, overall ORs of SYMPTOMS and ECG were 3.32 (95% CI: [2.72, 4.04]) (*P* < 0.0001) and 2.59 (95% CI: [2.14, 3.15]) (*P* < 0.0001), respectively. Subgroup analysis also found statistical significance in the differences between GXN treatment group and control group in testing GXN monotherapy and adjunctive therapy. These results indicated that GXN treatment is effective in treating angina pectoris. 

 The results of this meta-analysis were robust as shown in subgroup analysis, sensitivity analysis, and metaregression on various parameters including sample sizes, follow-up periods, daily dosages of GXN, types of angina pectoris, and the quality scores of RCTs. Although funnel plots, Begg's test, Egger's test, and trim-and-fill method found publication biases, the overall effects would still favor GXN treatment after enough number of less favorable studies were published to restore the symmetry of funnel plots. 

 The efficacy of GXN in both monotherapy and adjunctive therapy of angina pectoris exemplifies potential uses of chemical components of GXN as one of the herbal products that have offered great potentials in developing multitarget agents to treat complex diseases [91]. Experimental studies also showed that the aqueous extracts from both Danshen and Chuanxiong significantly reduced the myocardial infarct size in rat myocardial ischemia/reperfusion injury [92]. As seen from the clinical and experimental findings, GXN seems to be a promising resource for identifying new therapeutic agents or new drug targets [93] in treating angina pectoris. Although subgroup analysis and sensitivity analysis did not suggest any significant factors which would influence the efficacy of GXN, clinical heterogeneity may contribute to heterogeneity of this meta-analysis.

 The limitations of this study include small sample sizes and short follow-up periods. The mean sample size was 93, which was lower than 124 as required by an alpha of 0.05, the proportions of 0.899 for GXN and 0.742 for control group, and a power of 0.8 [94]. The patients of angina pectoris would need long-term treatment [95], but most available RCTs have short follow-up periods. 

 Another major limitation of this systematic review is the low quality of included studies although most of included RCT reports achieved the average quality of Chinese RCTs [96, 97], which is still inadequate. Almost all (63 out of 65) studies scored 2 at the Jadad scale, which ranges between 0 and 5. One study [34] reported single blinding and another study [47] reported double blinding. Twenty-four RCTs scored 4 at the *M* scale and 40 RCTs scored less than 4 at the *M* scale. There is evidence of the Cochrane Library's tool to show high risks of bias with the aspects of selection bias, performance bias, and detection bias. More than that, less than but almost half of included RCTs (28/65) did not report adverse events, one possible reason of which is high reporting bias for selecting reporting. Safety of GXN intervention cannot be assessed because of incomplete reporting data. Despite the fact that subgroup analysis found no statistically significant differences in ORs of SYMPTOMS and ECG between the RCTs of low and medium *M* scores, high-quality RCTs would be necessary to further support the efficacy of GXN-based medicines over conventional Western drugs in treating angina pectoris.

 Seventy-three out of 6064 participants had AE. The main AEs included headache (19), skin ecchymosis (14), epigastria discomfort (12), and palpitation (11). Headache was the most frequent AE in this paper. The AE mechanisms of GXN are not clear and definite. The functions of dilated blood vessels and coronary artery blood circulation activating are possible reasons that lead to adverse events.

 According to this meta-analysis, GXN seems to be effective in treating angina pectoris. As GXN contains the herbal extracts from Salvia miltiorrhiza and Ligustrazine, hence DSS, PAC, PAL, CAA, and SAB as the main active ingredients with potential effects on coronary heart disease, angina pectoris, and cardiovascular diseases [98] by enhancing coronary blood flow, improving the myocardial systolic functions, and protecting myocardial cells [99], further clinical, herbal formulation and pharmacological studies are warranted for further research and development. 

## 5. Conclusion

This meta-analysis of eligible RCTs provides evidence that GXN is effective in treating angina pectoris. This evidence warrants further RCTs of higher quality, longer follow-up periods, larger sample sizes, and multicentres/multicountries for more extensive subgroup, sensitivity, and metaregression analyses. 

## Supplementary Material

Supplementary Table 1 provides the treatment (drugs and dosages for control and treatment groups) details about the RCTs included in this study.

## Figures and Tables

**Table 1:** Characteristics of the included studies.

Study	Number of authors	Trial date report	Sample size	Followup (day)	Baseline comparison	AE	Outcomes measure	Treatment group dosage	Angina
Chen 2009	1	1	100	15	1	0	SYM, ECG	GXN 20 mL/d + CG	Angina
Chen et al. 2011	3	1	100	10	0	0	SYM	GXN 20 mL/d + CG	Angina
Chen 2006	1	1	62	14	1	1	SYM, ECG	GXN 20 mL/d + CG	Angina
Cheng and Zang 2010	2	1	43	14	1	0	SYM	GXN 30 mL/d + CG	Unstable
Cheng et al. 2011	3	1	76	14	1	1	SYM, ECG	GXN 30 mL/d + CG	Angina
Dong XP 2009	1	0	100	1	0	0.5	SYM	GXN 20 mL/d	Angina
Fu and Meng 2011	2	0	47	10	1	0	SYM, ECG	GXN 20 mL/d + CG	Angina
Fu et al. 2010	4	1	56	14	1	0	SYM, ECG	GXN 200 ml + CG + shenmaiyin 40 ml	Angina
Gao et al. 2005	3	1	60	14	1	1	SYM, ECG	GXN 20 mL/d	Angina
Gong et al. 2009	3	1	85	14	1	1	SYM, ECG	GXN 20 mL/d + xueshuantong 20 ml	Stable
He and Meng 2007	1	1	49	15	1	0	SYM	GXN 20 mL/d + CG	Unstable
He 2009	1	1	120	28	1	1	SYM, ECG	GXN 30 mL/d + atorvastatin 10 mg	Unstable
Hou and Gao 2009	2	1	128	14	1	1	SYM, ECG	GXN 20 mL/d + CG	Stable
Huang et al. 2011	4	0	120	7	1	1	SYM	GXN 20 mL/d + CG + xueshuantong 400 mg	Angina
Jiang et al. 2010	3	1	116	10	1	0	SYM, ECG	GXN 20 mL/d + CG	Unstable
Jiang 2009	1	1	68	20	1	1	SYM, ECG	GXN 20 mL/d	Angina
Jiang et al. 2010	5	0	56	7	0	1	SYM	GXN 30 mL/d	Angina
Kong 2009	1	0	100	14	1	1	SYM	GXN 30 mL/d + CG	Unstable
Lan et al. 2006	3	1	64	14	1	1	SYM	GXN 20 mL/d	Angina
Li and Jia 2011	2	1	200	14	1	0	SYM, ECG	GXN 30 mL/d + CG	Angina
Li and Lei 2005	2	1	156	14	0	1	SYM, ECG	GXN 20 mL/d + CG	Angina
Li et al. 2009	5	1	168	14	1	0	SYM	GXN 20 mL/d + CG	Angina
Li and Ran 2009	2	1	160	10	1	1	SYM	GXN 20 mL/d + CG	Angina
Li 2004	1	0	83	7	1	0	SYM, ECG	GXN 20 mL/d + CG	Unstable
Liang and Feng 2010	2	0	120	14	1	0	SYM, ECG	GXN 20 mL/d + CG	Unstable
Liu 2004	1	1	104	10	1	1	SYM	GXN 20 mL/d + CG	Unstable
Liu and Li 2007	2	1	88	12	0	1	SYM	GXN 20 mL/d + CG	Unstable
Liu 2005	1	1	80	30	1	0	SYM, ECG	GXN 20 mL/d + CG	Unstable
Liu 2011	1	1	152	28	1	0	SYM, ECG	GXN 20 mL/d + CG	Angina
Lu et al. 2006	3	1	68	30	1	0	SYM	GXN 30 mL/d + CG	Angina
Ma and Peng 2008	2	1	120	14	1	0	SYM	GXN 30 mL/d + CG	Unstable
Nie and Chen 2007	2	1	60	14	1	0	SYM, ECG	GXN 20 mL/d + CG	Angina
Qiao and Wu 2004	2	0	81	28	1	1	SYM, ECG	GXN 20 mL/d + CG	Stable
Song 2010	1	1	82	7	0	0.5	SYM	GXN 20 mL/d + CG + diltiazem 90 mg/d	Unstable
Su 2009	1	1	90	15	0	1	SYM, ECG	GXN 6 mL/d + CG	Angina
Sun 2010	1	1	90	14	1	0	SYM, ECG	GXN 30 mL/d + CG	Unstable
Sun et al. 2006	5	1	98	15	0	0	SYM	GXN 20 mL/d + CG	Angina
Tian and Wu 2006	2	1	62	14	1	1	SYM	GXN 30 mL/d + CG	Unstable
Wan and Xu 2009	2	0	120	14	1	1	SYM, ECG	GXN 30 mL/d + CG	Unstable
Wang 2007	1	1	100	14	1	1	SYM, ECG	GXN 30 mL/d + CG	Angina
Wang 2011	1	1	85	14	1	1	SYM, ECG	GXN 20 mL/d + CG	Unstable
Wang 2011	2	1	112	14	1	1	SYM, ECG	GXN 20 mL/d + CG	Unstable
Wang and Ji 2008	2	1	60	14	0	0	SYM, ECG	GXN 20 mL/d + CG	Unstable
Wang and Sun 2007	2	1	92	10	0	0	SYM	GXN 20 mL/d + CG	Unstable
Wang 2005	2	1	60	15	1	0	SYM	GXN 20 mL/d + CG	Unstable
Wang 2010	1	0	80	14	1	1	SYM, ECG	GXN 20 mL/d + CG	Stable
Wang 2005	1	0	76	15	0	0	SYM	GXN 20 mL/d + CG + shenmai 30 mL/d + tongxinluo 9 pills/d	Unstable
Wang 2005	1	1	60	14	1	1	SYM	GXN 20 mL/d + CG	Unstable
Wang et al. 2011	4	1	60	14	1	1	SYM	GXN 20 mL/d + CG	Unstable
Wu et al. 2008	3	0	108	14	1	0	SYM	GXN 20 mL/d + CG	Angina
Wu et al. 2011	4	1	144	7	1	0	SYM	GXN 20 mL/d + CG + shenmai 50 mL/d	Unstable
Xia 2011	1	1	90	14	1	0	SYM, ECG	GXN 30 mL/d + CG	Unstable
Yang and Ma 2008	2	1	90	14	1	0	SYM, ECG	GXN 30 mL/d + CG	Unstable
Ye et al. 2008	3	0	76	15	0	1	SYM	GXN 20 mL/d + CG	Unstable
Yu and Wang 2009	2	1	75	15	1	0	SYM, ECG	GXN 20 mL/d + CG	Angina
Yuan 2005	1	0	104	14	1	0	SYM, ECG	GXN 20 mL/d + CG	Angina
Zhang 2005	1	1	60	14	1	0	SYM, ECG	GXN 10 mL/d	Angina
Zhang 2010	1	1	240	15	1	1	SYM, ECG	GXN 20 mL/d + CG	Unstable
Zhang 2004	1	1	102	14	1	1	SYM, ECG	GXN 10 mL/d + CG + ginkgo leaf injection 10 mL/d	Angina
Zhang 2004	1	1	42	7	0	1	SYM	GXN 20 mL/d + CG	Angina
Zhao et al. 2010	6	1	100	14	1	1	SYM, ECG	GXN 10 mL/d + CG + xueshuangtong 120 mg	Unstable
Zhao and An 2008	2	1	90	28	1	1	SYM, ECG	GXN 20 mL/d + CG + simvastatin 10–20 mg/d	Unstable
Zhao 2010	1	1	86	14	1	0	SYM, ECG	GXN 30 mL/d + CG	Angina
Zhong et al. 2007	8	1	60	10	0	0	SYM	GXN 20 mL/d + CG	Angina
Zhu 2005	1	1	80	15	0	0.5	SYM, ECG	GXN 20 mL/d + CG	Unstable

GXN is Guanxinning injection; LMWH is low molecular weight heparin; and shenmai is Shenmai injection. CG is interventions of control group; SYM is SYMPTOMS; ECG is electrocardiogram; and AE is adverse event. The column of “Trial date report” shows that study did (1) or did not (0) report the trial date. The column of “Baseline comparison” shows that the study did (1) or did not (0) report the baseline comparison between the treatment and control groups.

**Table 2:** Quality assessment of included studies.

Study	C1	C2	C3	C4	C5	C6	C7	C8	Comparable	Random	Blind	Dropout	AE	Jadad	*M *
Chen 2009	Low	High	High	High	Low	Low	High	High	1	1	0	1	0	2	3
Chen et al. 2011	Low	Unclear	High	High	Low	Low	High	High	0	1	0	1	0	2	2
Chen 2006	Low	High	High	High	Low	Low	Low	Low	1	1	0	1	1	2	4
Cheng and Zeng 2010	Low	High	High	High	Low	Low	High	High	1	1	0	1	0	2	3
Cheng et al. 2011	Low	High	High	High	Low	Low	Low	Low	1	1	0	1	1	2	4
Dong 2009	Low	High	High	High	Low	Low	Unclear	High	0	1	0	1	0.5	2	2.5
Fu and Meng 2011	Low	High	High	High	Low	Low	High	High	1	1	0	1	0	2	3
Fu et al. 2010	Low	High	High	High	Low	Low	High	High	1	1	0	1	0	2	3
Gao et al. 2005	Low	Low	High	High	Low	Low	Low	Low	1	1	1	1	1	3	5
Gong et al. 2009	Low	High	High	High	Low	Low	Low	Low	1	1	0	1	1	2	4
He 2007	Low	High	High	High	Low	Low	High	High	1	1	0	1	0	2	3
He 2009	Low	High	High	High	Low	Low	Low	Low	1	1	0	1	1	2	4
Hou and Gao 2009	Low	High	High	High	Low	Low	Low	Low	1	1	0	1	1	2	4
Huang et al. 2011	Low	High	High	High	Low	Low	Low	Low	1	1	0	1	1	2	4
Jiang et al. 2010	Low	High	High	High	Low	Low	High	High	1	1	0	1	0	2	3
Jiang 2009	Low	High	High	High	Low	Low	Low	Low	1	1	0	1	1	2	4
Jiang et al. 2010	Low	High	High	High	Low	Low	Low	Low	0	1	0	1	1	2	3
Kong 2009	Low	High	High	High	Low	Low	Unclear	Low	1	1	0	1	1	2	4
Lan et al. 2006	Low	High	High	High	Low	Low	Low	Low	1	1	0	1	1	2	4
Li and Jia 2011	Low	High	High	High	Low	Low	High	High	1	1	0	1	0	2	3
Li and Lei 2005	Low	High	High	High	Low	Low	Low	Low	0	1	0	1	1	2	3
Li et al. 2009	Low	Low	Low	Low	Low	Low	High	High	1	1	2	1	0	4	5
Li and Ran 2009	Low	High	High	High	Low	Low	Low	Low	1	1	0	1	1	2	4
Li 2004	Low	High	High	High	Low	Low	High	Low	1	1	0	1	0	2	3
Liang and Feng 2010	Low	High	High	High	Low	Low	High	High	1	1	0	1	0	2	3
Liu 2004	Low	High	High	High	Low	Low	Low	Low	1	1	0	1	1	2	4
Liu and Li 2007	Low	High	High	High	Low	Low	High	High	0	1	0	1	1	2	3
Liu 2005	Low	High	High	High	Low	Low	High	High	1	1	0	1	0	2	3
Liu 2011	Low	High	High	High	Low	Low	High	High	1	1	0	1	0	2	3
Lu et al. 2006	Low	High	High	High	Low	Low	High	High	1	1	0	1	0	2	3
Ma and Peng 2008	Low	High	High	High	Low	Low	Low	Low	1	1	0	1	0	2	3
Nie and Chen 2007	Low	High	High	High	Low	Low	High	High	1	1	0	1	0	2	3
Qiao and Wu 2004	Low	High	High	High	Low	Low	Low	Low	1	1	0	1	1	2	4
Song 2010	Low	High	High	High	Low	Low	Low	Unclear	0	1	0	1	0.5	2	2.5
Su 2009	Low	Unclear	High	High	Low	Low	Low	Low	0	1	0	1	1	2	3
Sun 2010	Low	High	High	High	Low	Low	High	High	1	1	0	1	0	2	3
Sun et al. 2006	Low	High	High	High	Low	Low	High	Unclear	0	1	0	1	0	2	2
Tian and Wu 2006	Low	High	High	High	Low	Low	Low	Low	1	1	0	1	1	2	4
Wan and Xu 2009	Low	High	High	High	Low	Low	Low	Low	1	1	0	1	1	2	4
Wang 2007	Low	High	High	High	Low	Low	Low	High	1	1	0	1	1	2	4
Wang 2011	Low	High	High	High	Low	Low	Low	Low	1	1	0	1	1	2	4
Wang 2011	Low	High	High	High	Low	Low	Low	Low	1	1	0	1	1	2	4
Wang and Ji 2008	Low	High	High	High	Low	Low	High	High	0	1	0	1	0	2	2
Wang and Sun 2007	Low	High	High	High	Low	Low	High	Unclear	0	1	0	1	0	2	2
Wang 2005	Low	High	High	High	Low	Low	High	High	1	1	0	1	0	2	3
Wang 2010	Low	High	High	High	Low	Low	Low	Low	1	1	0	1	1	2	4
Wang 2005	Low	High	High	High	Low	Low	High	Unclear	0	1	0	1	0	2	2
Wang 2005	Low	Low	Unclear	High	Low	Low	Low	Low	1	1	0	1	1	2	4
Wang et al. 2011	Low	High	High	High	Low	Low	Low	Low	1	1	0	1	1	2	4
Wu et al. 2008	Low	High	High	High	Low	Low	High	Unclear	1	1	0	1	0	2	3
Wu et al. 2011	Low	High	High	High	Low	Low	High	High	1	1	0	1	0	2	3
Xia 2011	Low	Low	Unclear	High	Low	Low	High	Unclear	1	1	0	1	0	2	3
Yang and Ma 2008	Low	Low	Unclear	High	Low	Low	High	High	1	1	0	1	0	2	3
Ye et al. 2008	Low	High	High	High	Low	Low	Low	Low	0	1	0	1	1	2	3
Yu and Wang 2009	Low	High	High	High	Low	Low	High	High	1	1	0	1	0	2	3
Yuan 2005	Low	High	High	High	Low	Low	High	High	1	1	0	1	0	2	3
Zhang 2005	Low	High	High	High	Low	Low	High	High	1	1	0	1	0	2	3
Zhang 2010	Low	High	High	High	Low	Low	Low	Low	1	1	0	1	1	2	4
Zhang 2004	Low	High	High	High	Low	Low	Low	Low	1	1	0	1	1	2	4
Zhang 2004	Low	High	High	High	Low	Low	Low	High	0	1	0	1	1	2	3
Zhao et al. 2010	Low	High	High	High	Low	Low	Low	Low	1	1	0	1	1	2	4
Zhao and An 2008	Low	High	High	High	Low	Low	Low	Low	1	1	0	1	1	2	4
Zhao 2010	Low	High	High	High	Low	Low	High	Unclear	1	1	0	1	0	2	3
Zhong et al. 2007	Low	High	High	High	Low	Low	High	High	0	1	0	1	0	2	2
Zhu 2005	Low	High	High	High	Low	Low	Unclear	Unclear	0	1	0	1	0.5	2	2.5

C1 is random sequence generation for selection bias; C2 is allocation concealment for selection bias; C3 is blinding of participants and personnel for performance bias; C4 is blinding of outcome assessment (patient-reported outcomes) for detection bias; C5 is blinding of outcome assessment (SYMPTOMS) for detection bias; C6 is incomplete outcome data addressed for attrition bias; C7 is reporting bias for selecting reporting; C8 is other sources of bias for other bias; Comparable is participants in treat group and control group comparable; Random is study described as randomized; Blind is study described as blinding; Dropout is withdrawals and dropouts of participants; AE is the adverse effects; Low is low risk of bias; High is high risk of bias; Unclear is unclear risk of bias.

**Table 3:** Subgroups and sensitivity analysis on SYMPTOMS outcomes.

	Group	Number of RCTs	Number of participants	OR	Wilcoxon test	95% CI	Z	*P* (eff)	*I* ^2^	*χ* ^2^	*P* (het)
*M* score	≤3	40	3625	3.21	*W* = 546	2.36, 4.35	7.46	<0.0001	54%	0.50	<0.0001
	>3	25	2439	3.51	*P* = 0.5395	2.78, 4.43	10.50	<0.0001	0%	0	0.9858
Sample size	<93	39	2772	3.22	*W* = 445.5	2.59, 4.01	10.51	<0.0001	0%	0	0.6150
	≥93	26	3292	3.37	*P* = 0.4140	2.39, 4.76	6.89	<0.0001	60%	0.47	<0.0001
Number of authors	1	27	2485	3.18	*W* = 1189	2.39, 4.24	7.92	<0.0001	28%	0.16	0.1253
	>1	38	3579	3.40	*P* = 0.7558	2.60, 4.46	8.87	<0.0001	44%	0.30	0.0031
Publication year	≤2008	31	2495	3.80	*W* = 441.5	3.01, 4.81	11.19	<0.0001	1%	0.01	0.2929
	>2008	34	3569	2.94	*P* = 0.2642	2.20, 3.93	7.32	<0.0001	48%	0.34	0.0016
Trial date report	Reported	51	4793	3.19	*W* = 2112.5	2.57, 3.95	10.52	<0.0001	36%	0.21	0.0189
	Not reported	14	1271	3.84	*P* = 1	2.33, 6.33	5.28	<0.0001	47%	0.40	0.0254
Baseline comparison	Reported	50	4808	3.56	*W* = 2112.5	2.84, 4.45	11.10	<0.0001	40%	0.25	0.0057
	Not reported	15	1256	2.53	*P* = 1	1.75, 3.68	4.89	<0.0001	14%	0.08	0.1545
Adverse events	Reported	31	2947	3.20	*W* = 1006	2.58, 3.97	10.59	<0.0001	0%	0	0.4304
	Not reported	34	3117	3.48	*P* = 0.4678	2.53, 4.78	7.68	<0.0001	51%	0.44	0.0003
Follow-up period (day)	≤14	48	4461	3.38	*W* = 440	2.75, 4.16	11.51	<0.0001	28%	0.14	0.1321
	>14	17	1603	3.05	*P* = 0.6382	1.81, 5.16	4.18	<0.0001	61%	0.71	0.0005
GXN daily	6–200 mL	65	6064	3.32	*W* = 2059	2.72, 4.04	11.93	<0.0001	37%	0.23	0.0030
Dosage (mL)	6–30 mL	64	6008	3.34	*P* = 0.9231	2.73, 4.07	11.83	<0.0001	38%	0.24	0.0025
GXN daily	<20	4	352	3.42	*χ* ^2^ = 0.4290	1.48, 7.91	2.88	0.0040	38%	0.28	0.1717
Dosage (mL)	20	45	4235	3.16	df = 2	2.45, 4.07	8.85	<0.0001	46%	0.33	0.0004
	>20	16	1477	3.87	*P* = 0.8069	2.84, 5.29	8.51	<0.0001	0%	0	0.8315
Types of angina	Stable	4	374	3.42	*χ* ^2^ = 0.9900	1.89, 6.21	4.05	<0.0001	0%	0	0.7151
	Unstable	31	2892	3.07	df = 2	2.26, 4.16	7.18	<0.0001	47%	0.34	0.0013
	Angina	30	2798	3.61	*P* = 0.6096	2.72, 4.81	8.81	<0.0001	32%	0.19	0.1179
Improvement	>50%	65	6064	3.32	*W* = 02.5	2.72, 4.04	11.93	<0.0001	37%	0.23	0.0030
	>80%	63	5856	1.75	*P* < 0.0001	1.54, 1.98	8.65	<0.0001	25%	0.06	0.0557
GXN	1	6	408	3.19	*χ* ^2^ = 0.4891	1.86, 5.49	4.21	<0.0001	0%	0	0.8454
GXN + CG	2	49	4681	3.43	df = 2	2.81, 4.19	12.07	<0.0001	21%	0.11	0.1177
GXN + CG + additional	3	10	975	3.07	*P* = 0.7830	1.47, 6.41	2.99	0.00228	72%	0.98	<0.0001

CI is confidence interval; *Z* and *P* (eff) are statistical terms for evaluating overall effect; *I* ^2^, *χ* ^2^, and *P* (het) are statistical terms for assessing heterogeneity among studies.

**Table 4:** Subgroups and sensitivity analysis on ECG outcomes.

	Group	Number of RCTs	Number of participants	OR	Wilcoxon test	95% CI	*Z *	*P* (eff)	*I* ^2^	*χ* ^2^	*P* (het)
*M* score	≤3	21	1995	2.47	*W* = 149	1.79, 3.41	5.53	<0.0001	52%	0.28	0.0025
	>3	17	1709	2.71	*P* = 0.3945	2.17, 3.39	8.77	<0.0001	0%	0	0.9136
Sample size	<93	23	1734	2.42	*W* = 127	1.93, 3.02	7.72	<0.0001	0%	0	0.9776
	≥93	15	1970	2.86	*P* = 0.1789	1.94, 4.21	5.33	<0.0001	67%	0.37	0.0002
Number of authors	1	19	1872	2.30	*W* = 140	1.72, 3.08	5.65	<0.0001	41%	0.16	0.0358
	>1	19	1832	2.98	*P* = 0.2428	2.34, 3.80	8.81	<0.0001	13%	0.04	0.4435
Publication year	≤2008	15	1268	2.49	*W* = 200	1.94, 3.20	7.17	<0.0001	0%	0	0.9538
	>2008	23	2436	2.68	*P* = 0.4200	1.99, 3.61	6.47	<0.0001	52%	0.26	0.0025
Trial date report	Reported	31	3069	2.43	*W* = 57	1.97, 3.00	8.29	<0.0001	34%	0.12	0.0511
	Not reported	7	635	3.67	*P* = 0.0548	2.36, 5.70	5.79	<0.0001	8%	0.03	0.5363
Baseline comparison	Reported	34	3318	2.64	*W* = 85	2.14, 3.24	9.20	<0.0001	35%	0.12	0.0513
	Not reported	4	386	2.29	*P* = 0.4325	1.25, 4.19	2.68	0.0074	23%	0.09	0.2200
Adverse events	Reported	19	1955	2.67	*W* = 192	2.16, 3.30	9.14	<0.0001	0%	0	0.8792
	Not reported	19	1749	2.54	*P* = 0.7480	1.80, 3.59	5.28	<0.0001	54%	0.31	0.0020
Follow-up period (day)	≤14	27	2528	2.83	*W* = 129	2.34, 3.42	10.67	<0.0001	2%	0	0.7120
	>14	11	1176	2.21	*P* = 0.5407	1.37, 3.57	3.27	<0.0001	65%	0.39	0.0024
GXN daily	6–200 mL	38	3704	2.59	*W* = 707.5	2.14, 3.15	9.68	<0.0001	32%	0.11	0.0539
dosage (mL)	6–30 mL	37	3648	2.58	*P* = 0.9662	2.12, 3.14	9.45	<0.0001	33%	0.1175	0.0448
GXN daily	<20	4	352	1.89	*χ* ^2^ = 3.4288,	1.00, 3.55	1.96	0.0497	27%	0.1148	0.2425
dosage (mL)	20	24	2324	2.80	df = 2	2.14, 3.66	7.51	<0.0001	43%	0.1820	0.0246
	>20	10	1028	2.53	*P* = 0.1801	1.85, 3.46	5.81	<0.0001	14%	0.0365	0.5413
Types of angina	Stable	4	374	3.03	*χ* ^2^ = 0.7010	1.80, 5.09	4.18	<0.0001	0%	0	0.6688
	Unstable	16	1676	2.48	df = 2	1.95, 3.15	7.42	<0.0001	10%	0.02	0.2332
	Angina	18	1654	2.60	*P* = 0.7043	1.87, 3.61	5.68	<0.0001	46%	0.22	0.0191
Improvement	>50%	38	3704	2.59	*W* = 1050	2.14, 3.15	9.68	<0.0001	32%	0.11	0.0539
	>80%	38	3704	1.84	*P* < 0.0001	1.59, 2.14	8.06	<0.0001	0%	0	0.8367
GXN	1	3	188	3.15	*χ* ^2^ = 1.6604	1.71, 5.81	3.68	0.0002	0%	0	0.9202
GXN + CG	2	29	2963	2.68	df = 2	2.10, 3.41	7.98	<0.0001	42%	0.17	0.0157
GXN + CG + additional	3	6	553	2.09	*P* = 0.4360	1.45, 3.01	3.94	<0.0001	0%	0	0.6382

CI is confidence interval; *Z* and *P* (eff) are statistical terms for evaluating overall effect; *I* ^2^, *χ* ^2^, and *P* (het) are statistical terms for assessing heterogeneity among studies.

**Table 5:** Metaregression analysis of the relationship between outcomes and the study characteristics.

log OR	Number of RCTs	Number of participants	Factor	Coefficient	*z *	*P *
SYMPTOMS	65	6064	*M* score	0.0663	0.4378	0.6615
			Sample size	−0.0013	−0.4955	0.6203
			Number of authors	−0.0466	−0.6283	0.5298
			Publication year	−0.0838	−1.9158	0.0554
			Trial date report	−0.1931	−0.7634	0.4453
			Baseline comparison	0.3299	1.3376	0.1810
			Adverse events	−0.0965	−0.4646	0.6422
			Follow-up period	0.0116	0.6126	0.5401

ECG	38	3704	*M* score	0.1191	0.7160	0.4740
			Sample size	0.0006	0.2938	0.7689
			Number of authors	−0.0100	−0.1071	0.9147
			Publication year	−0.0180	−0.4296	0.6675
			Trial date report	−0.4255	−1.5606	0.1186
			Baseline comparison	0.1520	0.4458	0.6558
			Adverse events	0.1066	0.5300	0.5961
			Follow-up period	−0.0423	−2.6000	0.0093

**Table 6:** Adverse events reported in the included studies.

	Treatment group		Control group	
	Number of AEs	Number of studies	Number of AEs	Number of studies
Headache	10	4	9	3
Dizziness	1	1	1	1
Palpitation	4	2	7	3
Skin ecchymosis	8	2	6	1
Serum transaminase elevated	1	1	NR	NR
Nausea	1	1	3	1
Epigastria discomfort	4	2	8	3
Abnormal liver function	1	1	NR	NR
Skin allergy	NR	NR	1	1
General weakness	NR	NR	1	1
Cold sweat	NR	NR	5	1
Hypotension	NR	NR	1	1
Skin mucosal bleeding	NR	NR	1	1
No AEs	0	27	0	24
Total AEs reports	30	9	43	11
No AE report	0	28	0	29

NR: not reported; AEs: adverse events.

## References

[1] Gaziano TA (2005). Cardiovascular disease in the developing world and its cost-effective management. *Circulation*.

[2] Kung HC, Hoyert DL, Xu JQ, Murphy SL (2008). Deaths: final data for 2005. National Vital Statistics Reports. *National Center for Health Statistics*.

[3] Scottish Intercollegiate Guidelines Networks (2007). *Management of Stable Angina. A National Clinical Guideline*.

[4] Tan SL, Liu CL (2001). Clinical observation of compound danshen dripping pill treating stable angina pectoris. *Heilongjiang Medical Journal*.

[5] Chen XF, Lou ZY, Zhang H (2011). Identification of multiple components in Guanxinning injection using hydrophilic interaction liquid chromatography/time-of-flight mass spectrometry and reversed-phase liquid chromatography/time-of-flight mass spectrometry. *Rapid Communications in Mass Spectrometry*.

[6] Cheng TO (2007). Cardiovascular effects of danshen. *International Journal of Cardiology*.

[7] Sun YY, Li SF, Quan C (2005). Solubility of ferulic acid and tetramethylpyrazine in supercritical carbon dioxide. *Journal of Chemical and Engineering Data*.

[8] Chen HP (2009). Efficacy analysis of Guanxinning injection combined with isosorbide mononitrate treating ischemic heart disease. *Chinese Journal of Ethnomedicine and Ethnopharmacy*.

[9] Shen YL, Sang ZL (2011). Efficacy analysis of Guanxinning injection treating ischemic heart disease. *World Health Digest Medical Periodieal*.

[10] Fu XY, Zhao XH (2009). Clinical analysis of Guanxinning injection treating ischemic heart disease or cerebral infarction. *China Modern Medicine*.

[11] Moher D, Liberati A, Tetzlaff J, Altman DG (2009). The PRISMA Group. Preferred reporting items for systematic reviews and meta-analyses: the PRISMA Statement. *Plos Medicine*.

[12] Wang JM (2011). Meta-analysis of Guanxinning injection as adjunctive therapy for unstable Angina pectoris. *China Pharmacy*.

[13] Jadad AR, Moore RA, Carroll D (1996). Assessing the quality of reports of randomized clinical trials: is blinding necessary?. *Controlled Clinical Trials*.

[14] Jia Y, Huang F, Zhang S, Leung SW (2012). Is danshen (Salvia miltiorrhiza) dripping pill more effective than isosorbide dinitrate in treating angina pectoris? A systematic review of randomized controlled trials. *International Journal of Cardiology*.

[15] Higgins J, Green S (2008). Analyzing data and undertaking meta-analyses. *Cochrane Handbook For Systematic Reviews of Interventions Version 5.1.0*.

[16] (1979). Nomenclature and criteria for diagnosis of ischemic heart disease. Report of the Joint International Society and Federation of Cardiology/World Health Organization Task Force on standardization of clinical nomenclature. *Circulation*.

[17] Lewis T PROC LOGISTIC: the logistics behind interpreting categorical variable effects.

[18] Breierova L, Choudhari M (2001). An introduction to sensitivity analysis. *Massachusetts Institute of Technology*.

[19] Higgins JPT, Thompson SG (2002). Quantifying heterogeneity in a meta-analysis. *Statistics in Medicine*.

[20] Sterne JAC, Egger M (2001). Funnel plots for detecting bias in meta-analysis: guidelines on choice of axis. *Journal of Clinical Epidemiology*.

[21] Begg CB, Mazumdar M (1994). Operating characteristics of a rank correlation test for publication bias. *Biometrics*.

[22] Egger M, Smith GD, Schneider M, Minder C (1997). Bias in meta-analysis detected by a simple, graphical test. Increase in studies of publication bias coincided with increasing use of meta-analysis. *British Medical Journal*.

[23] Duval SJ, Tweedie RL (2000). Trim and fill: a simple funnel-plot-based method of testing and adjusting for publication bias in meta-analysis. *Biometrics*.

[24] Thompson SG, Higgins JPT (2002). How should meta-regression analyses be undertaken and interpreted?. *Statistics in Medicine*.

[25] R Development Core Team R: a language and environment for statistical computing. http://www.r-project.org/.

[26] Chen HP (2009). Clinical observation of Guanxinning combined with isosorbide mononitrate in the treatment of Coronary Heart Disease. *Chinese Journal of Ethnomedicine and Ethnopharmacy*.

[27] Chen RJ, Yang X, Yang XS (2011). Clinical observation of Guanxinning combined with potassium magnesium aspartate in the treatment of coronary artery disease. *Contemporary Medicine*.

[28] Chen SG (2006). Clinical observation of Guanxinning injection in the treatment of coronary artery disease. *Modern Journal of Integrated Traditional Chinese and Western Medicine*.

[29] Cheng HY, Zhang WP (2010). The clinical experience with Guanxinning injection in the treatment of elderly diabetic patients with unstable angina. *Journal of Bingtuan Medicine*.

[30] Cheng YS, Tan RB, Zhao DM (2011). Guanxinning injection in the treatment of unstable angina pectoris: a report of 72 cases. *China Foreign Medical Treatment*.

[31] Dong XP (2009). Clinical observation of Guanxinning injection in the treatment of angina pectoris: a report of 60 cases. *Chinese Journal of Modern Drug Application*.

[32] Fu YC, Meng LQ (2011). Clinical observation of Guanxinning injection in the treatment of angina pectoris. *Journal of New Chinese Medicine*.

[33] Fu YW, Jiao CX, Shi YH, Guo F (2010). Clinical observation of Guanxinning injection combined with Shengmai injection in the treatment of Coronary Heart Disease. *Journal of Emergency in Traditional Chinese Medicine*.

[34] Gao GQ, Wang F, Sun HP (2005). Clinical observation of Guanxinning injection in the treatment of angina pectoris. *Nei Mongol Journal of Traditional Chinese Medicine*.

[35] Gong CJ, Wang PJ, Huang LM (2009). Clinical observation of Xueshuantong combined with Guanxinning injection in the treatment of angina pectoris. *Asia-Pacific Traditional Medicine*.

[36] He HY (2007). Clinical observation of Guanxinning injection in the treatment of unstable angina: a report of 27 cases. *China Modern Doctor*.

[37] He YJ (2009). Clinical observation of atorvastatin combined with Guanxinning injection in the treatment of unstable angina. *Chinese Journal of Clinical Rational Drug Use*.

[38] Hou GP, Gao XY (2009). Clinical observation of Guanxinning injection and dipyridamole in the treatment of angina pectoris. *Journal of Qiqihar Medical College*.

[39] Huang Y, Li B, Li DH, Chen JS (2011). Clinical observation on low molecular weight heparins calcium combined with Guanxinning and Xuesaitong in cerebral infarction. *Journal of Clinical Rational Drug Use*.

[40] Jiang S, Xiong ZY, Yong FZ (2010). Clinical observation of Guanxinning injection in the treatment of unstable angina pectoris. *China Modern Doctor*.

[41] Jiang SH (2009). Clinical observation of Guanxinning injection in the treatment of unstable angina: a report of 38 cases. *China Medical Herald*.

[42] Jiang SL, Zheng SY, Guo XS, Yi SH, Zou XY (2010). Clinical observation of Guanxinning injection in the treatment of angina pectoris. *China Foreign Medical Treatment*.

[43] Kong LX (2009). Clinical observation of Guanxinning injection combined with ferulic sodium in the treatment of unstable angina pectoris. *Clinical Medicine*.

[44] Lan PM, Luo WB, Weng GM (2006). Clinical observation of Guanxinning Injection in the treatment of coronary artery disease and effects on blood lipids. *Chinese Medicine Modern Distance Education of China*.

[45] Li CT, Jia XZ (2011). Clinical observation of Guanxinning injection in the treatment of diabetic patients with angina pectoris: a report of 100 cases. *Chinese Journal of Clinical Healthcare*.

[46] Li H, Lei JP (2005). Guanxinning Injection in the treatment of angina pectoris. *Journal of Medical Forum*.

[47] Li H, Cai H, Wang LZ, Yang HY, Ma DM (2009). The improvement of heart function of Guanxinning injection in the patients with angina decubitus. *China Medical Herald*.

[48] Li L, Ran GX (2009). Clinical observation Guanxinning Injection adjuvant therapy for angina pectoris: a report of 100 cases. *Shandong Medical Journal*.

[49] Li XB (2004). Clinical observation of low molecular weight heparin combined with Guanxinning injection in the treatment of unstable angina. *Heilongjiang Medical Journal*.

[50] Liang HY, Feng YG (2010). Clinical observation of low molecular weight heparin combined with Guanxinning injection in the treatment of unstable angina. *Medical Innovation of China*.

[51] Liu BQ (2004). Clinical observation of low molecular weight heparin and Guanxinning injection in the treatment of unstable angina. *Medicine Industry Information*.

[52] Liu L, Li MZ (2007). Clinical observation Guanxinning Injection in the treatment of angina pectoris: a report of 48 cases. *Jiangxi Journal of Traditional Chinese Medicine*.

[53] Liu YL (2005). Clinical observation of Guanxinning injection in the treatment of unstable angina pectoris. *Chinese Journal of Integrated Traditional and Western Medicine in Intensive and Critical Care*.

[54] Liu ZH (2011). Qi decoction in the treatment of angina pectoris: a report of 80 cases. *Journal of Traditional Chinese Medicine*.

[55] Lu CX, Zhang J, Tang B (2006). Guanxinning injection combined with isosorbide dinitrate treating the aged patients with ischemic heart disease angina pectoris. *Journal of Yangtze University (Nature Science Edition)*.

[56] Ma XY, Peng LW (2008). Clinical observation of Guanxinning injection in the treatment of unstable angina. *Journal of Modern Clinical Medicine*.

[57] Nie YB, Chen HY (2007). Clinical observation of Guanxinning injection in the treatment of angina pectoris. *Journal of Huaihai Medicine*.

[58] Qiao WL, Wu ZR (2004). Guanxinning injection for stable angina pectoris of coronary heart disease. *Journal of Henan University of Chinese Medicine*.

[59] Song GF (2010). Analysis study of Diltiazem combined with Guanxinning in the treatment of unstable angina pectoris. *Chronic Pathematology Journal*.

[60] Su XD (2009). Clinical observation Guanxinning Injection in the treatment of angina pectoris and myocardial ischemia. *Proceeding of Clinical Medicine*.

[61] Sun SP (2010). Clinical efficacy analysis of Guanxinning combined with Western medicine in the treatment of unstable angina. *Jinlin Medical Journal*.

[62] Sun ZH, Dong PF, Sun DW, Zhao LY (2006). Guanxinning injection in the treatment of angina pectoris: a report of 50 cases. *Harbin Medical Journal*.

[63] Tian ZQ, Wu L (2006). Clinical observation of Guanxinning combined with Western medicine in the treatment of unstable angina. *Chinese Journal of Cardiovascular Rehabilitation Medicine*.

[64] Wan SQ, Xu AY (2009). Clinical observation of Guanxinning injection in the treatment of unstable angina pectoris. *Modern Journal of Integrated Traditional Chinese and Western Medicine*.

[65] Wang E (2007). Clinical observation of Guanxinning injection in the treatment of angina pectoris. *Journal of Emergency in Traditional Chinese Medicine*.

[66] Wang GL (2011). Clinical observation of Guanxinning injection in the treatment of unstable angina pectoris: a report of 43 cases. *Chinese Remedies and Clinics*.

[67] Wang HT, Yan CM (2011). Clinical observation of Guanxinning injection in the treatment of unstable angina pectoris. *Chinese Journal of Clinical Research*.

[68] Wang JJ, Ji XP (2008). Clinical observation of Guanxinning injection in the treatment of unstable angina pectoris. *Journal of Liaoning University of TCM*.

[69] Wang LJ, Sun XM (2007). Clinical observation of Guanxinning injection in the treatment of unstable angina pectoris. *Journal of Qiqihar Medical College*.

[70] Wang Q (2005). Clinical observation of Guanxinning injection in the treatment of angina pectoris. *Modern Medicine and Health*.

[71] Wang Q (2010). Clinical observation of Guanxinning injection in the treatment of stable angina pectoris: a report of 40 cases. *Yunnan Journal of Traditional Chinese Medicine and Materia Medica*.

[72] Wang RZ (2005). Clinical observation of Guanxinning combined with Western medicine in the treatment of unstable angina: a report of 38 cases. *The Journal of Medical Theory and Practice*.

[73] Wang Y (2005). Clinical observation of Guanxinning injection treating ischemic heart disease angina pectoris. *Modern Medicine Health*.

[74] Wang ZB, Li HJ, Zhang XY, Li SQ (2011). Clinical observation of Guanxinning injection in the treatment of unstable angina pectoris. *Chinese Community Doctors*.

[75] Wu XF, Wang YF, Liu JP (2008). Clinical observation of guanxinning injection in the treatment of coronary heart disease and angina. *China Foreign Medical Treatment*.

[76] Wu YG, Zhao S, Fan XJ, Zhao WQ (2011). Guanxinning onjection combined with Shenmai injection for unstable angina pectoris: a report of 72 cases. *Journal of Anhui TCM College*.

[77] Xia Y (2011). Clinical observation of Guanxinning combined with Western medicine in the treatment of unstable angina: a report of 50 cases. *Shaanxi Journal of Traditional Chinese Medicine*.

[78] Yang T, Ma ZX (2008). Clinical observation of Guanxinning combined with Western medicine in the treatment of unstable angina. *Asia-Pacific Traditional Medicine*.

[79] Ye XW, Luo YS, Gui F (2008). Clinical observation of Low molecular weight heparin combined with Guanxinning injection in the treatment of unstable angina. *Zhejiang Journal of Integrated Traditional Chinese and Western Medicine*.

[80] Yu HJ, Wang WF (2009). Clinical observation of Guanxinning injection combined with nitroglycerin in the treatment of angina pectoris: a report of 40 cases. *Nei Mongol Journal of Traditional Chinese Medicine*.

[81] Yuan L (2005). Clinical observation of Guanxinning injection in the treatment of angina pectoris. *Henan Traditional Chinese Medicine*.

[82] Zhang LX (2005). Guanxinning injection in the treatment of angina pectoris: a report of 35 cases. *Zhejiang Journal of Traditional Chinese Medicine*.

[83] Zhang LX (2010). Analysis study of Guanxinning injection in the treatment of unstable angina pectoris: a report of 120 cases. *China Clinical Practical Medicine*.

[84] Zhang Y (2004). Clinical observation of Guanxinning combined with Ginkgo biloba in the treatment of unstable angina. *Journal of Changzhi Medical College*.

[85] Zhang ZX (2004). Nitroglycerin combined with Guanxinning injection in the treatment of unstable angina. *Zhejiang Journal of Integrated Traditional Chinese and Western Medicine*.

[86] Zhao FL, Lu XB, Gong CJ, Wang PJ, Huang LM, Guo K (2010). Clinical observation of Guanxinning injection combined with Xueshuantong in the treatment of unstable angina. *Journal of Practical Traditional Chinese Medicine*.

[87] Zhao PT, An Y (2008). Clinical observation of Guanxinning Injection combined with simvastatin in the treatment of unstable angina pectoris. *Contemporary Medicine*.

[88] Zhao YJ (2010). Clinical observation of Guanxinning in the treatment of angina: a report of 43 cases. *Chinese Journal of Integrated Traditional and Western Medicine in Intensive and Critical Care*.

[89] Zhong TH, Li W, Liang YD (2007). Clinical observation of Guanxinning in the treatment of angina: a report of 35 cases. *Chinese Medicine Modern Distance Education of China*.

[90] Zhu L (2005). Integrated traditional and Western treatment of unstable angina: a report of 80 cases. *Forum on Traditional Chinese Medicine*.

[91] Wang L, Zhou GB, Liu P (2008). Dissection of mechanisms of Chinese medicinal formula Realgar-Indigo naturalis as an effective treatment for promyelocytic leukemia. *Proceedings of the National Academy of Sciences of the United States of America*.

[92] Zhang DW, Liu JG, Feng JT (2010). Effects of effective components compatibility of aqueous extracts of Salviae Miltiorrhizae and Rhizoma Chuanxiong on rat myocardial ischemia/reperfusion injury. *Zhongguo Wei Zhong Bing Ji Jiu Yi Xue*.

[93] Li XJ, Zhang HY (2008). Synergy in natural medicines: implications for drug discovery. *Trends in Pharmacological Sciences*.

[94] Wang D, Bakhai A (2006). *Clinical Trials: A Practical Guide to Design, Analysis, and Reporting*.

[95] Gheorghiade M, Bonow RO (1998). Chronic heart failure in the United States: a manifestation of coronary artery disease. *Circulation*.

[96] Tang JL, Zhan S, Ernst E (1999). Review of randomized controlled trials of traditional Chinese medicine. *British Medical Journal*.

[97] Liu J, Kjaergard LL, Gluud C (2002). Misuse of randomization: a review of Chinese randomized trials of herbal medicines for chronic hepatitis B. *American Journal of Chinese Medicine*.

[98] Guo X, Chen X, Li L (2008). LC-MS determination and pharmacokinetic study of six phenolic components in rat plasma after taking traditional Chinese medicinal-preparation: guanxinning lyophilized powder for injection. *Journal of Chromatography B*.

[99] Yang SM, Deng GP (2007). The evolvement in clinical use of Perhexiline injection. *Modern Hospital*.

