# Supplementary material for: The Efficacy of Guanxinning Injection in Treating Angina Pectoris: Systematic Review and Meta-Analysis of Randomized Controlled Trials
Source: Evid Based Complement Alternat Med. 2013 Mar 24;2013:282707. doi: 10.1155/2013/282707 (PMC3619549; doi:10.1155/2013/282707)
Supplement: Supplementary file 1 — Supplementary Table 1 provides the treatment (drugs and dosages for control and treatment groups) details about the RCTs included in this study. [file 282707.f1.pdf]

Supplementary Table 1. Drug dosage of the included studies.

Study	Treatment group dosage	Control group (CG) dosage
Chen HP, 2009	GXN+CG	Isosorbide mononitrate 20mg qd, polarized liquid with intravenous drip, aspirin 75mg qd, simvastatin 20mg qd
Chen RJ, 2011	GXN+CG	Danshen injection 20ml qd, simvastatin, entericcoated aspirin
Chen SG, 2006	GXN+CG	Beta blockers, nitrate esters, statin cholesterol drugs, aspirin 100mg qd
Cheng HY, 2010	GXN+CG	Bayaspirin, beta blockers, nitrates
Cheng YS, 2011	GXN+CG	Entericcoated aspirin 150mg qd, simvastatin 20mg qd, nitrates, beta blockers, ACEI
Dong XP, 2009	GXN	Nitroglycerin 10-50g qd
Fu YC, 2011	GXN+CG	Aspirin, beta blockers, nitrate esters
Fu YW, 2010	GXN+CG+additional	Conventional Western medicine
Gao GQ, 2004	GXN	Nitroglycerin 10g qd
Gong CJ, 2009	GXN+CG+additional	Puerarin
He HY, 2007	GXN+CG	ISDN 10mg tid, isosorbide mononitrate 10mg tid, aspirin 75mg
He YJ, 2009	GXN+CG+additional	Danshen injection 30ml, entericcoated aspirin, beta blockers, ACEI, CCB
Hou GP, 2009	GXN+CG	Dipyridamole injection 30mg qd, entericcoated aspirin 75mg qd, betaloc 25-50mg bid, ISDN 10mg tid
Huang Y, 2011	GXN+CG	Xuesaitong capsule 400mg qd, aspirin 150mg qd, 20% mannitol 125ml with intravenous drip
Jiang S, 2010	GXN+CG	Aspirin 100mg qd, ACEI or ARB,beta blockers, simvastatin 20mg qd, LMWH, nitrate esters
Jiang SH, 2009	GXN	Huangqi injection 20ml qd
Jiang SL, 2010	GXN	Xuesaitong injection 500mg qd
Kong LX, 2009	GXN+CG	Puerarin 400-600mg qd, nitroglycerin 10-15mg, LMWH 4100u, aspirin 100mg qd, metoprolol 25mg bid, simvastatin 20mg qd
Lan PM, 2006	GXN	Polarized liquid with intravenous drip
Li CT, 2011	GXN+CG	Isosorbide mononitrate injection 20ml qd
Li H, 2005	GXN+CG	Danshen injection 16-20ml qd, ISDN, aspirin, nitroglycerin
Li H, 2009	GXN+CG	Nitrates, entericcoated aspirin, beta blockers, ACEI, CCB
Li L, 2009	GXN+CG	Huangqi injection 30ml/d, conventional treatment
Li XB, 2004	GXN+CG	Entericcoated aspirin 100 mg qd, betaloc 25 mg bid, ISDN 10mg tid
Liang HY, 2010	GXN+CG	Aspirin, betaloc,CCB, nitrate esters
Liu BQ, 2005	GXN+CG+additional	Aspirin, ISDN, polarized liquid, beta blockers, CCB
Liu L, 2007	GXN+CG	Nitrates, ACEI, beta blockers, heparin
Liu YL, 2005	GXN+CG	ISDN, betaloc
Liu ZH, 2011	GXN+CG	GXN 20ml/d, Yiqi tongmai soap, ISDN 10mg tid, entericcoated aspirin 75mg qd
Lu CX, 2006	GXN+CG	Isosorbide mononitrate injection 20ml bid

Ma XY, 2008	GXN+CG	Entericcoated aspirin 300mg qd, simvastatin 20mg qd, nitrates, beta blockers, ACEI
Nie YB, 2007	GXN+CG	Nitrates, beta blockers, CCB, coronary artery vasodilators, antiplatelet aggregating
Qiao WL, 2004	GXN+CG	Nitrates,aspirin
Song GF, 2010	GXN+CG+additional	Entericcoated aspirin, ISDN, plavix,atorvastatin
Su XD, 2009	GXN+CG	Danshen injection 20ml qd,, nitroglycerin 10mg
Sun SP, 2010	GXN+CG	LMWH, aspirin,nitroglycerin, ACEI, beta blockers
Sun ZH, 2006	GXN+CG	Aspirin, isosorbide mononitrate
Tian ZQ, 2006	GXN+CG	LMWH, aspirin, nitroglycerin, ACEI, beta blockers
Wan SQ, 2009	GXN+CG	LMWH, aspirin, nitroglycerin, ACEI, beta blockers
Wang E, 2007	GXN+CG	Aspirin, nitroglycerin, ACEI, beta blockers
Wang GL, 2011	GXN+CG	ECG monitoring, anticoagulant, antiplatelet aggregating, dilated coronary artery drug, lipid lowering treatment
Wang HT, 2011	GXN+CG	Aspirin 100mg/d,ISDN 10mg tid, metoprolol 6.25-12.5mg bid, enalapril 5mg bid, atorvastatin 20mg qd, polarized liquid
Wang JJ, 2008	GXN+CG	Beta blockers, nitrate esters, statin cholesterol drugs
Wang LJ, 2007	GXN+CG	ISDN 10mg tid, entericcoated aspirin 50mg qd
Wang Q, 2005	GXN+CG	Aspirin 75mg qd, nitroglycerin 10mg qd
Wang Q, 2010	GXN+CG	Danshen injection 20ml qd, beta blockers, ACEI, nitrates, statins, bayaspirin
Wang RZ, 2005	GXN+CG+additional	Nitroglycerin 10-15mg, hypodermic injection LMWH 4100u, aspirin 75-100mg qd, betaloc 25mg
Wang Y, 2005	GXN+CG	Xiangdan injection, ISDN, asporin, ACEI, beta blockers
Wang ZB, 2011	GXN+CG	Aspirin, ISDN, LMWH, ACEI,betaloc
Wu XF, 2008	GXN+CG	Danshen injection 10ml qd, conventional Western medicine
Wu YG, 2011	GXN+CG+additional	Aspirin, nitrates, statin, ACEI, beta blockers, LMWH
Xia Y, 2011	GXN+CG	LMWH, nitroglycerin, ACEI, beta blockers
Yang T, 2008	GXN+CG	LMWH, nitroglycerin, ACEI, beta blockers
Ye XW, 2008	GXN+CG	GXN 20ml, LMWH calium 5000U, beta blockers, nitrates, aspirin
Yu HJ, 2009	GXN+CG	Nitroglycerin 10mg qd, 10％ calcium chloratum l0mg, 25％ magnesium sulfate 10mg, insulin 8u, polarized liquid
Yuan L, 2005	GXN+CG	Nitrates,beta blockers, CCB, coronary artery vasodilators, antiplatelet aggregating, anticoagulant
Zhang LX, 2005	GXN	Nitroglycerin 0.9g qd
Zhang LX, 2010	GXN+CG	Nitrates, aspirin, antiplatelet aggregating
Zhang Y, 2004	GXN+CG+additional	Entericcoated aspirin 75mg qd, betaloc 25-50mg bid, ISDN 10mg tid, isosorbide mononitrate 20mg bid or tid
Zhang ZX, 2004	GXN+CG	Entericcoated aspirin, ISDN, nifedipine, low molecular dextran 500ml, Salvia miltiorrhiza needle 20ml
Zhao FL, 2010	GXN+CG+additional	Nitroglycerin 10-15mg, LMWH 4100U, entericcoated aspirin 100mg, betaloc 25mg
Zhao PT, 2008	GXN+CG+additional	Aspirin, CCB, ACEI, beta blockers
Zhao YJ, 2010	GXN+CG	Nitroglycerin 10mg, entericcoated aspirin 75-100mg

Zhong TH, 2007	GXN+CG	5% glucose 250ml, isoket 10mg
Zhu L, 2005	GXN+CG	ISDN 30mg/d, entericcoated aspirin, betaloc, LMWH

ACEI is angiotensin-converting enzyme inhibitors; CCB is calcium channel blockers; GXN is Guanxinning injection; ISDN is isosorbide dinitrate; LMWH is low molecular weight heparin; Shenmai: Shenmai injection; tid is three times per day; bid is two times per day; qd is once per day.
